# Evaluating Intact Parathyroid Hormone for Differentiating Chronic Kidney Disease From Acute Kidney Injury in Hospitalized Adults With Kidney Dysfunction

**DOI:** 10.7759/cureus.90518

**Published:** 2025-08-19

**Authors:** Juan Nicolas Dallos Ferrerosa, Natalia A Uribe, Alberto Sierra, Karen D Sibaja, Camilo A Ayala, Mariana Agudelo, Jose M. Ustariz, Ligia L Calderon, Fabian Jaimes, Joaquin Rodelo

**Affiliations:** 1 Internal Medicine, Universidad de Antioquia, Medellin, COL; 2 Medicine, Universidad de Antioquia, Medellin, COL; 3 Nephrology, Nephrology Unit, Hospital San Vicente Fundación, Medellin, COL; 4 Nephrology, Nephrology Unit, Hospital San Vicente Fundacion, Medellin, COL; 5 Internal Medicine, Universidad de Antioquia, Medellín, COL; 6 Nephrology, Nephrology Unit, Hospital San Vicente Fundación, Medellín, COL

**Keywords:** acute kidney injury, art of diagnosis, chronic kidney disease (ckd), kidney function, parathyroid hormone (pth)

## Abstract

Background*:* Distinguishing between chronic kidney disease (CKD) and acute kidney injury (AKI) in hospitalized patients without prior medical records is a common yet critical challenge, particularly in emergency settings, with direct implications for clinical decision-making and patient outcomes. This study aimed to evaluate the diagnostic performance of intact parathyroid hormone (iPTH) in differentiating AKI from CKD in adults admitted to the hospital with kidney dysfunction.

Methods: This retrospective cohort study included hospitalized adults (aged 18 years and older) with serum creatinine levels ≥1.3 mg/dL, no prior history of CKD, and available iPTH measurements, from January 2015 to July 2023. Two nephrologists, blinded to the iPTH results, independently classified patients as having CKD or AKI. In cases of disagreement, a third nephrologist provided the final classification. The diagnostic performance of iPTH was assessed using receiver operating characteristic (ROC) curves, and optimal cut-off values were determined using Bayes' theorem.

Results: A total of 200 patients were included (107 with AKI and 93 with CKD). The area under the ROC curve for iPTH in distinguishing AKI from CKD was 0.81 (95% CI 0.70-.090). Cut-off values of 125, 132, and 170 pg/mL yielded sensitivities of 75.3%, 71%, and 57%, and specificities of 73.8%, 77.6%, and 88.8%, respectively. Values ≥170 pg/mL were most specific for confirming CKD, while levels <125 pg/mL favored AKI.

Conclusion: iPTH effectively distinguishes AKI from CKD. A cut-off of 170 picograms per milliliter is optimal for confirming CKD, while values below 125 picograms per milliliter are useful for ruling it out.

## Introduction

Kidney disease is a significant public health challenge in Colombia and globally, with substantial economic implications for healthcare systems [1]. Its incidence rises with advancing population age. Kidney disease is categorized based on its progression as either acute kidney injury (AKI) or chronic kidney disease (CKD). According to the KDIGO (Kidney Disease: Improving Global Outcomes) guidelines, CKD is defined as an abnormality in kidney structure or function persisting for more than three months, irrespective of the underlying cause. Similarly, AKI is defined per KDIGO criteria as an abrupt decline in kidney function, encompassing conditions such as acute kidney failure [2-4].

In clinical practice, patients with impaired kidney function but no clear history of kidney disease are frequently encountered, and differentiating between AKI and CKD in such cases can be challenging. This distinction is crucial, particularly in the early, reversible stages of kidney disease, to guide timely interventions and prevent progression [5]. However, current diagnostic tools lack specificity [6-8], and in many cases, a kidney biopsy remains the only definitive method. Unfortunately, kidney biopsy is invasive, costly, associated with risks, and often unavailable [9].

The current understanding of kidney pathophysiology suggests that intact parathyroid hormone (iPTH) levels increase progressively across CKD stages, reaching levels two to three times above the upper normal limit [6]. In AKI, however, lower iPTH levels are expected since parathyroid hyperplasia and subsequent hormone elevation require time to develop. This makes iPTH a potential marker for assessing the chronicity of kidney dysfunction [10,11].

Although only one study has specifically addressed the role of iPTH in distinguishing AKI from CKD, the hormone is widely used in clinical practice and is recommended by several guidelines for monitoring kidney disease progression and represents a marker that may be useful in assessing the chronicity of kidney disease [10]. Given the limited research in this area, particularly regarding its discriminatory value between AKI and CKD, this study aimed to evaluate the utility of iPTH in distinguishing these two conditions and to propose a clinically applicable threshold for its use in hospitalized patients with no prior history of kidney disease.

## Materials and methods

Study design

This was an analytical, retrospective cohort study designed to evaluate a diagnostic test. The report follows the STARD statement for diagnostic accuracy studies [12].

Participants

Patients aged ≥18 years with impaired kidney function, defined as a serum creatinine level ≥1.3 mg/dL and no prior history of CKD, were included. The absence of CKD was confirmed by reviewing electronic medical records to verify previous estimated glomerular filtration rate (eGFR) values > 60 mL/min/1.73 m² before hospitalization. Exclusion criteria were patients who died within 24 hours of hospital admission; those with obstructive uropathy; missing data (i.e., no iPTH levels, no creatinine result, and/or no imaging available to assess cortical and kidney size); patients who received outpatient care or were not hospitalized; those referred to another institution; patients with SARS-CoV-2 infection; those taking medications known to affect iPTH levels; those with acute kidney disease, defined as kidney injury persisting for more than seven days, and individuals with a history of parathyroid disease or parathyroid surgery.

Patients were identified through the registry of iPTH test requests in the electronic medical records system of the Hospital Universitario San Vicente Fundación (HUSVF), a high-complexity institution located in Medellín, Colombia. The study period spanned from January 1, 2015, to July 31, 2023.

The study protocol was reviewed and approved by the Ethics Committee of HUSVF (Acta número 35-2022) and the Research Committee of Antioquia University (Universidad de Antioquia).

Test description

Serum iPTH was measured using the Atellica™ IM Intact Parathyroid Hormone assay (Siemens Healthineers, Erlangen, Germany), a non-competitive two-point immunoassay employing direct chemiluminescent technology. This method utilizes constant amounts of two anti-human PTH antibodies. The Lite Reagent consists of a mouse monoclonal anti-human PTH antibody (N-terminal) labeled with acridinium ester, while the solid phase includes a biotinylated (C-terminal) mouse monoclonal anti-human PTH antibody coupled to streptavidin-coated paramagnetic latex particles. The normal laboratory reference range for serum iPTH is 18.5 to 88.0 pg/mL (1.96 to 9.33 pmol/L).

The assay demonstrates laboratory precision with a standard deviation of ≤1.0 pg/mL (0.1 pmol/L) for samples <10.0 pg/mL (1.1 pmol/L), a coefficient of variation (CV) ≤10% for samples between 10.0 - 20.0 pg/mL (1.1 - 2.12 pmol/L), a CV ≤8% for samples between 20.0 and 700.0 pg/mL (2.12 - 74.2 pmol/L), and a CV ≤10% for samples >700.0 pg/mL (74.2 pmol/L) (data from the laboratory test insert).

In this study, the clinical evaluation and agreement of two nephrologists (LLC and JMU) were established as the reference standard. In cases of disagreement, a third nephrologist (JRR) resolved the discordance. Inter-rater agreement between the two primary reviewers was summarized as overall percent agreement with 95% confidence intervals (CI; Wilson method) and quantified using Cohen’s kappa (κ) with asymptotic 95% CI to account for chance agreement. The proportion of cases requiring adjudication was also reported. The nephrologists’ diagnoses were based on clinical history, patient evolution during hospitalization, the need for kidney replacement therapy (KRT), and laboratory tests, including creatinine behavior, blood urea nitrogen (BUN), calcium, phosphorus, hemoglobin, 25-OH vitamin D levels, 24-hour urine protein, kidney biopsy results (if performed), and anatomical features of kidney size and cortex observed via ultrasound or abdominal tomography. No rigid numerical cut-offs were applied for calcium or hemoglobin; values were interpreted in the context of other findings. Comorbidities (e.g., diabetes, hypertension, HIV) were noted but not used as standalone diagnostic criteria. The eGFR values at admission and discharge were calculated using the CKD-EPI 2021 formula [13]. All evaluators were blinded to the iPTH values.

Statistical analysis

Data for the study were extracted from the hospital's electronic medical records by trained independent evaluators. The data were entered into a database created using Microsoft Excel (Microsoft Corporation, Redmond, USA), with double-checking for accuracy. No imputation was made for missing data, and the frequency or central tendency values were reported with the number of available data points. There was no missing data in either the index or reference tests.

Descriptive statistics were calculated for quantitative variables, with normality of distributions assessed using the Shapiro-Wilk test. Normally distributed variables were presented as mean ± standard deviation (SD), while non-normally distributed variables were presented as median with interquartile range (IQR). Comparisons of iPTH values between patients with CKD and AKI were made using the Mann-Whitney U test (Wilcoxon), as the distribution of the variable did not meet the normality assumption.

A nonparametric analysis of the area under the ROC curve (AUC) [14] was performed with iPTH values as the independent variable and expert diagnoses as the reference variable, to determine the discriminatory capacity of the test. Using Bayes' theorem, the sensitivity, specificity, predictive values, and likelihood ratios were estimated at the cut-off point with the highest proportion of correctly classified patients (true positives and true negatives).

Sample size calculation was based on assumptions of 90% sensitivity, 90% specificity, and a 5% occurrence of CKD, with a 5% alpha error and 20% beta error. The required sample size was calculated to be 160 patients [1]. Statistical analysis was performed using STATA version 16 software (StataCorp, College Station, TX, USA).

## Results

Participants

**Figure 1:**
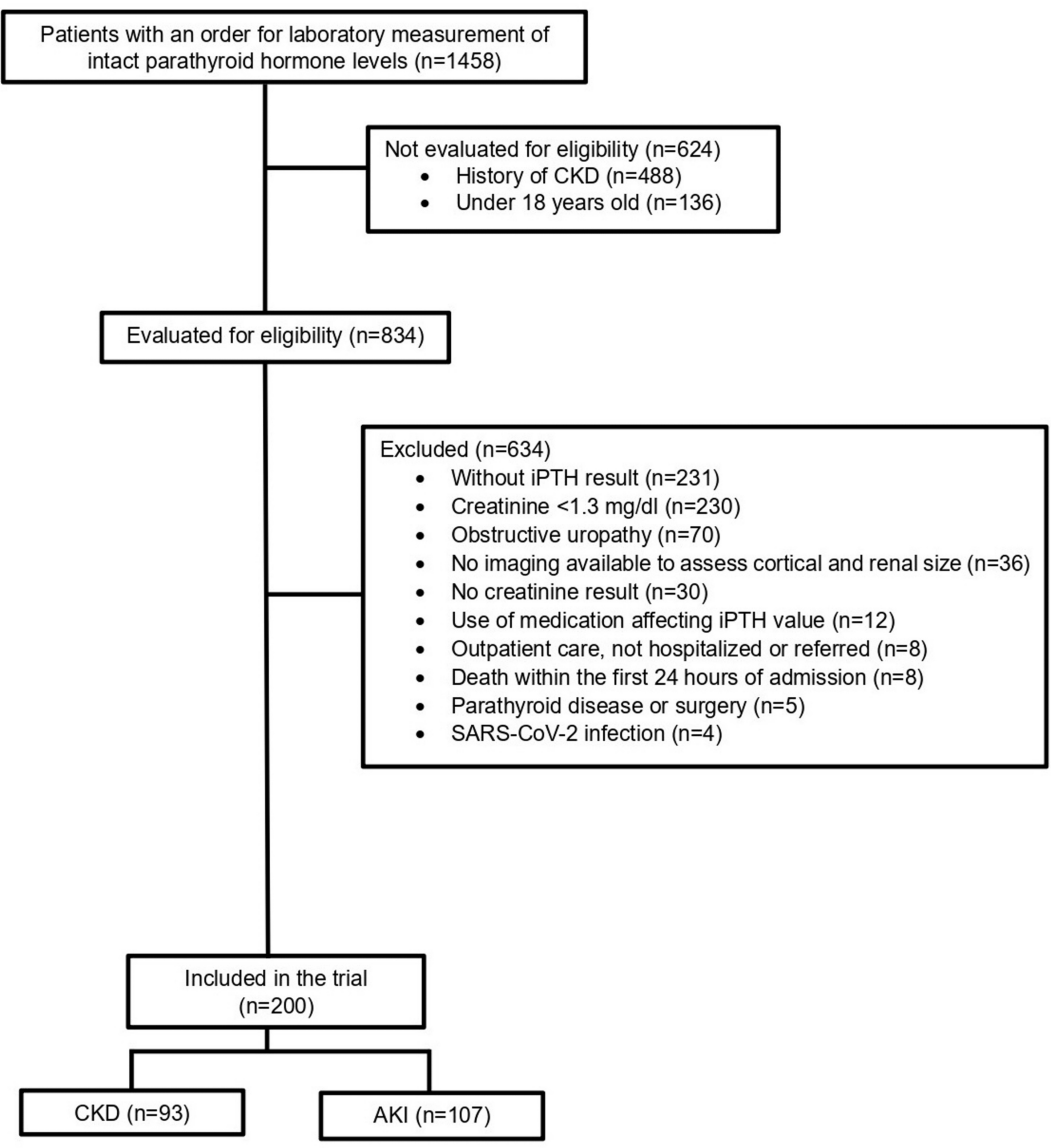
Flow diagram of patients enrolled in the study. AKI: acute kidney injury; CKD: chronic kidney disease; iPTH: intact parathyroid hormone.

Between January 1, 2015, and July 31, 2023, 1458 iPTH requests were reviewed at the clinical laboratory of the HUSVF in Medellín, Colombia. Of these, 624 were excluded due to ineligibility (patients younger than 18 years or with a history of CKD). The remaining 834 tests were reviewed, and 634 were excluded, predominantly due to missing iPTH results or creatinine levels <1.3 mg/dL. A total of 200 patients were included for analysis (Figure 1).

The demographic and clinical characteristics of the patients are summarized in Table 1. The median age was 63 years (IQR 50-74), and 122 (61%) of the patients were male. Most patients, 165 (82.5%), were admitted through the emergency department. Common comorbidities included arterial hypertension in 121 (60.5%) patients, diabetes mellitus in 58 (29%) patients, and a history of smoking in 42 (21%) patients. There was a notable difference in the history of heart failure, which was more frequent in the CKD group compared to the AKI group (18 (19.3%) vs. 10 (9.3%), respectively). 

**Table 1:** Demographic and clinical characteristics of hospitalized patients with kidney dysfunction according to the final diagnosis (CKD vs. AKI). Data presented in means and (standard deviation). BUN: blood urea nitrogen; COPD: chronic obstructive pulmonary disease; eGFR: estimated glomerular filtration rate; ICU: intensive care unit; iPTH: intact parathyroid hormone; IQR: interquartile range; SCU: special care unit; CKD: chronic kidney disease; AKI: acute kidney injury

Variable	Total (n=200)	Chronic (n=93)	Acute (n=107)	p
Age (years)	63 (50–74)	62 (49–74)	63 (52–74)	0.725
Male	122 (61%)	58 (62.4%)	64 (59.8%)	0.822
Female	78 (39%)	35 (37.6%)	43 (40.2%)	0.796
Emergency room	165 (82.5%)	75 (80.6%)	90 (84.1%)	0.647
Hospitalization	5 (2.5%)	3 (3.2%)	2 (1.9%)	0.665
ICU	26 (13%)	15 (16.1%)	11 (10.3%)	0.309
SCU	4 (2%)	0 (0%)	4 (3.7%)	0.124
Arterial hypertension	121 (60.5%)	63 (67.7%)	58 (54.2%)	0.070
Diabetes mellitus	58 (29%)	28 (30%)	29 (27.1%)	0.754
Smoking	42 (21%)	18 (19.3%)	24 (25.2%)	0.719
Heart failure	28 (14%)	18 (19.3%)	10 (9.3%)	0.067
Dyslipidemia	26 (13%)	5 (5.3%)	12 (11.2%)	0.221
COPD	17 (8.5%)	8 (8.6%)	9 (8.4%)	0.999
Coronary heart disease	14 (7%)	6 (6.4%)	8 (7.4%)	0.995
Obesity	11 (5.5%)	4 (4.3%)	8 (7.4%)	0.387
Cancer	8 (4%)	6 (6.4%)	5 (4.6%)	0.810
Cerebrovascular disease	4 (2%)	1 (1%)	3 (2.8%)	0.624
Creatinine at admission (mg/dL)	2.5 [1.7–4.5]	2.9 [2.1–6.9]	1.9 [1.6–3.1]	0.000
eGFR at admission (mL/min/1.73m²)	25 [12.0–38.5]	18 [8.0–32.0]	50 [19.0–42.0]	0.000
Creatinine at discharge (mg/dL)	1.7 [1.1–2.7]	2.5 [1.9–4.7]	1.1 [0.9–1.6]	0.001
eGFR at discharge (mL/min/1.73m²)	39.5 [22.7–69.0]	26.5 [13.0–35.7]	67.5 [42.5–0.7]	0.001
BUN at admission (mg/dL)	45.0 [30.4–69.8]	54.4 [33.0–87.7]	41.0 [28.9–65.2]	0.022
Hemoglobin (g/dL)	11.5 [9.1–13.0]	10.8 [9.1–12.0]	11.8 [9.3–13.3]	0.141
iPTH (pg/mL)	122.3 [67.5–198.8]	183.5 [125.4–288.9]	77.3 [51.4–126.8]	0.000
Serum calcium (mg/dL)	8.2 [7.6–8.7]	8.1 [7.6–8.6]	8.2 [7.7–9.0]	0.086
Phosphorus (mg/dL)	4.4 [3.4–5.5]	4.9 [3.6–6.2]	3.9 [3.1–5.0]	0.000
Albumin (g/dL)	3.2 (0.6)	3.2 (0.6)	3.2 (0.6)	0.999
Vitamin D (ng/mL)	21.3 [15.4–27.0]	21.1 [14.9–25.4]	22.6 [15.9–33.1]	0.009
24-hour urinary protein (g)	1.2 [0.6–3.9]	2.9 [0.7–4.5]	0.9 [0.5–1.5]	0.082
Cortical size (mm)	8.2 [7–10]	7.0 [7–10]	9.0 [7–10]	0.000
Right cortical size (mm)	9.1 [7–10]	7.0 [7–10]	9.0 [7–10]	0.000
Left cortical size (mm)	8.9 [7–10]	8.0 [7–10]	9.0 [7–11]	0.003
Kidney length (mm)	101.0 (15.6)	95.2 (17.3)	103.9 (18.8)	0.001
Right kidney length (mm)	101.0 (15.6)	95.9 (15.5)	103.6 (19.3)	0.010
Left kidney length (mm)	101.0 (15.8)	94.5 (18.9)	104.3 (10.5)	0.000
Kidney biopsy - Performed	16 (8%)	11 (11.8%)	5 (4.7%)	0.09
Kidney biopsy - Not performed	184 (92%)	82 (88.2%)	102 (95.3%)	0.109
Intrahospital dialysis - Yes	68 (34%)	40 (43%)	28 (26.2%)	0.018
Intrahospital dialysis - No	132 (66%)	53 (57%)	79 (73.8%)	0.018
Indication to dialysis - Yes	31 (15.5%)	24 (25.8%)	7 (6.5%)	0.001
Indication to dialysis - No	163 (81.5%)	65 (69.9%)	98 (91.6%)	0.001
Indication to dialysis - Unknown	6 (3%)	4 (4.3%)	2 (1.9%)	0.001

The two primary reviewers agreed in 181/200 (90.5%) cases (95% CI 85.6%-93.8%). Cohen’s kappa was 0.81 (95% CI 0.67-0.95), indicating almost perfect agreement. By final diagnosis subgroup, agreement was 94/107 (87.9%) for AKI (95% CI 80.3%-92.8%) and 87/93 (93.5%) for CKD (95% CI 86.6%-97.0%). A total of 19/200 (9.5%) cases (95% CI 6.2%-14.4%) required adjudication by the third reviewer.

Serum creatinine values at admission and discharge for patients with CKD were 2.9 mg/dL (IQR 2.1-6.9) and 2.5 mg/dL (IQR 1.9-4.7), respectively. These values were higher compared to the AKI group, which had admission values of 1.9 mg/dL (IQR 1.6-3.1) and discharge values of 1.1 mg/dL (IQR 0.9-1.6). Kidney size was smaller in patients with CKD, with an average length of 95.2 mm (SD 17.3), compared to 103.9 mm (SD 18.8) in the AKI group.

The iPTH value was higher in patients with CKD (183.5 pg/mL) compared to those with AKI (77.3 pg/mL), with a significant difference (p = 0.001). A higher number of patients requiring dialysis both in-hospital and at discharge was observed in those with higher iPTH levels. Kidney biopsy, which was rarely performed, was more common in patients with CKD than in those with AKI (11.8% vs. 4.7%, respectively).

**Figure 2:**
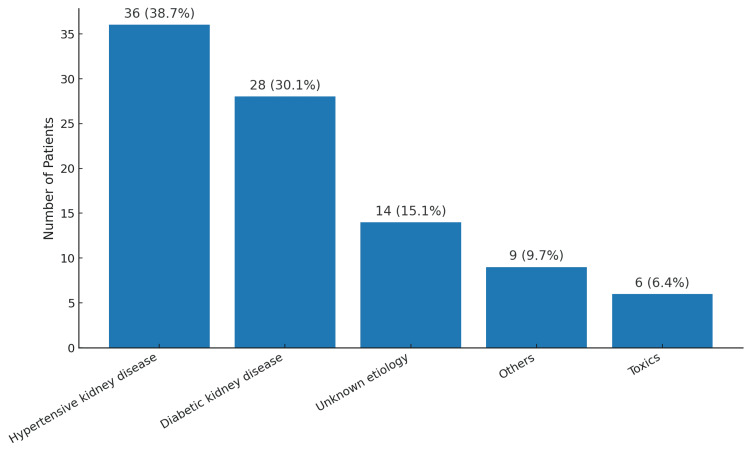
Main etiologies of chronic kidney disease.

**Figure 3:**
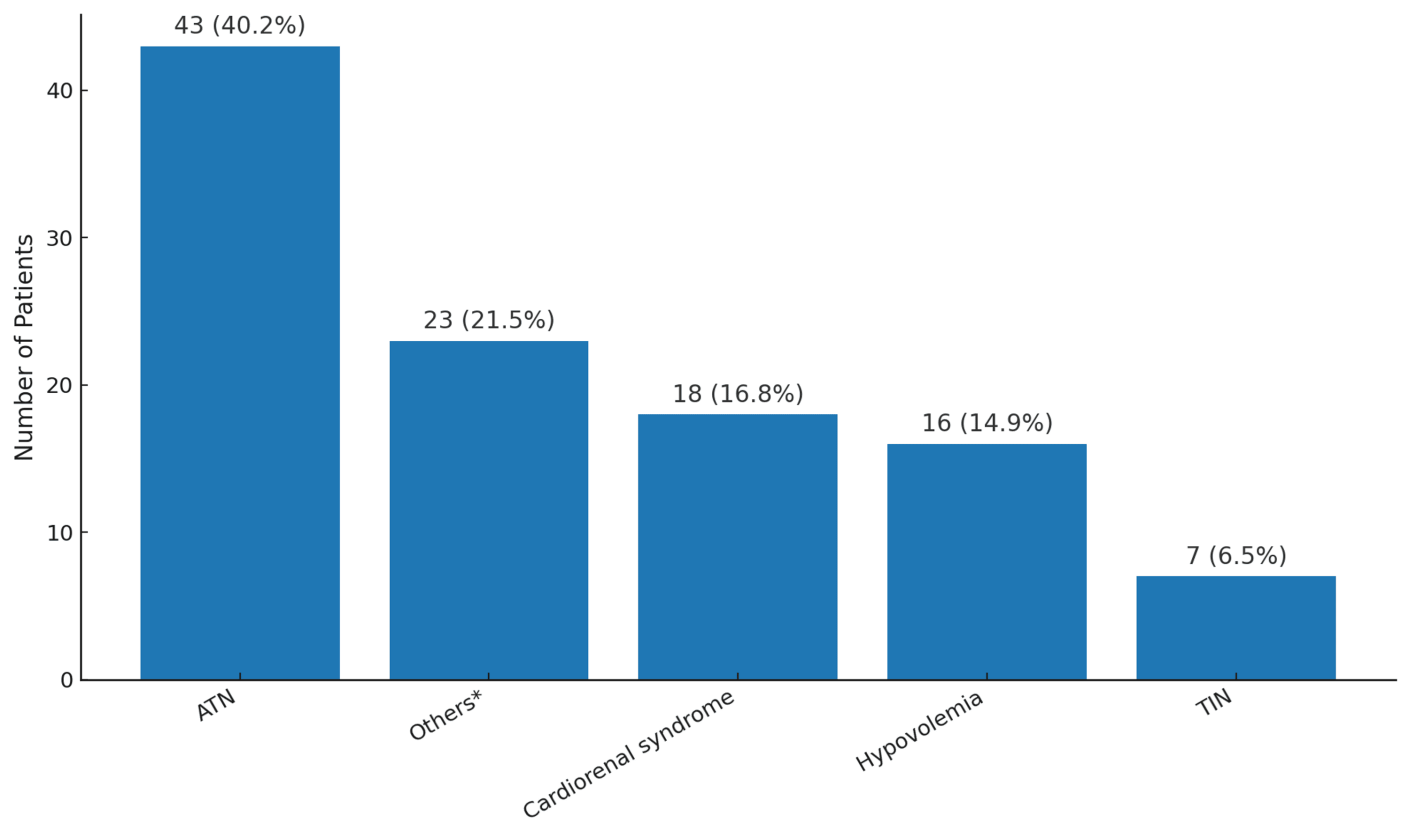
Main etiologies of acute kidney injury. ATN: acute tubular necrosis; TIN: tubulointerstitial nephritis. *Others includes multiple myeloma (n=4), acute pulmonary edema (n=1), diabetic ketoacidosis (n=1), hypertensive emergency (n=2), pulmonary embolism (n=2), and snakebite envenomation (n=2). See Table 2 for full details.

The primary etiologies of CKD included hypertensive kidney disease (36; 38.7%) and diabetic kidney disease (28; 30.1%), while acute tubular necrosis (43; 40.2%) and cardiorenal syndrome (18; 16.8%) were the leading causes of AKI (Figures 2, 3).

Test results

**Figure 4:**
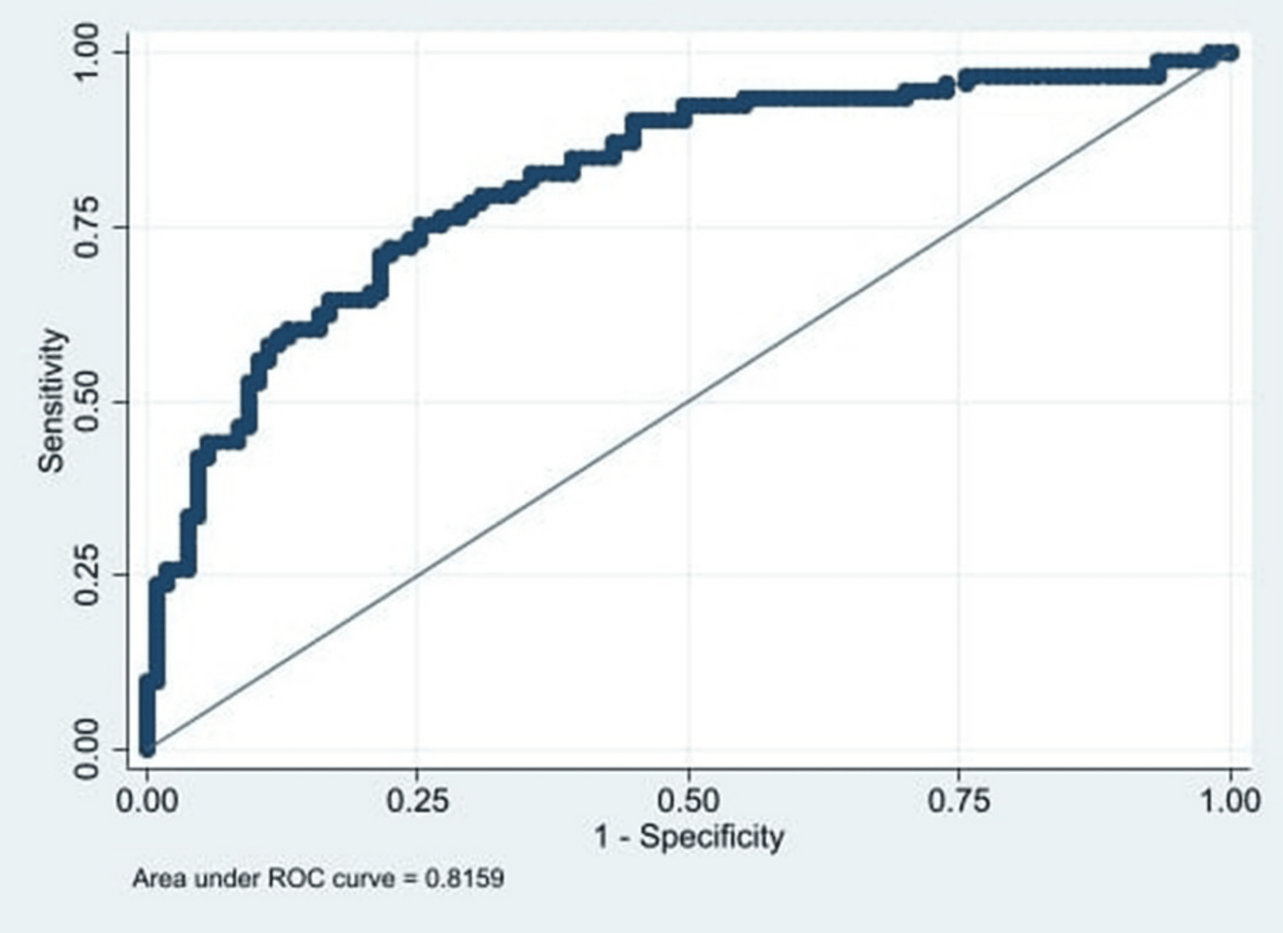
Area under the ROC curve for the diagnosis of chronic kidney disease using iPTH values. iPTH: intact parathyroid hormone, 95% CI 0.7 – 0.9.

The AUC for iPTH to differentiate CKD from AKI was 0.81 (95% CI 0.7 - 0.9) (Figure 4).

At a cut-off value of 170 pg/mL, iPTH demonstrated a sensitivity of 57% (95% CI 46.9 - 67%) and a specificity of 88.8% (95% CI 82.8 - 94.8%) (Table 2).

**Table 2:** Operating characteristics of different iPTH values. iPTH: intact parathyroid hormone; AUC: area under the curve; LR: likelihood ratio; OR: odds ratio; PPV: positive predictive value; NPV: negative predictive value

iPTH Value	125 pg/mL	132 pg/mL	170 pg/mL
Sensitivity	75.3%	71.0%	57.0%
Specificity	73.8%	77.6%	88.8%
AUC	0.7	0.7	0.7
LR+	2.9	3.2	5.1
LR−	0.3	0.4	0.5
OR	8.6	8.4	10.5
PPV	71.4%	73.3%	81.5%
NPV	77.5%	75.5%	70.4%

Additional cut-off points of 125 pg/mL and 132 pg/mL were associated with the highest percentage of correctly classified patients (75%), with corresponding sensitivity and specificity values (Table 3).

**Table 3:** Operational characteristics of different iPTH values. iPTH: intact parathyroid hormone; LR: likelihood ratios

iPTH Value (pg/mL)	Sensitivity	Specificity	Correctly Classified	LR+	LR−
122.5	76.34%	72.90%	74.50%	2.81	0.32
125.0	75.30%	73.80%	75.00%	2.88	0.33
126.0	74.19%	74.77%	74.50%	2.94	0.34
128.0	73.12%	75.70%	74.50%	3.00	0.35
131.3	72.04%	76.64%	74.50%	3.08	0.36
132.0	71.00%	77.60%	75.00%	3.16	0.37
132.5	70.97%	77.57%	74.50%	3.16	0.37
133.6	69.89%	78.50%	74.50%	3.25	0.38
143.4	64.52%	83.18%	74.50%	3.83	0.42
160.0	60.22%	86.92%	74.50%	4.60	0.45
165.0	59.14%	87.85%	74.50%	4.86	0.46
170.0	57.00%	88.80%	74.50%	5.08	0.48

For 125 pg/mL, the sensitivity was 75.3% (95% CI 66.5 - 84%) and specificity was 73.8% (95% CI 65.5 - 82.2%), while for 132 pg/mL, sensitivity was 71% (95% CI 61.7 - 80.2%) and specificity was 77.6% (95% CI 69.7 - 83.1%). The cut-off point of 125 pg/mL had the fewest false negatives (23 patients), while 170 pg/mL had the fewest false positives (12 patients) (Table 4).

**Table 4:** Contingency table: classification of kidney disease according to iPTH cut-off points. iPTH: Intact parathyroid hormone *125 pg/ml: 28 false positives and 23 false negatives; ** 132 pg/ml: 24 false positives and 27 false negatives; *** 170 pg/ml 12 false positives and 40 false negatives

iPTH Cut-off	Chronic Kidney Disease by iPTH Value	Final Diagnosis: CKD Yes	Final Diagnosis: CKD No	Total
125 pg/mL*	Yes	70	23	93
	No	28	79	107
	Total	98	102	200
132 pg/mL**	Yes	66	27	93
	No	24	83	107
	Total	90	110	200
170 pg/mL***	Yes	53	40	93
	No	12	95	107
	Total	65	135	200

## Discussion

The AUC for iPTH in this study was 0.81, indicating that the test performs well in distinguishing AKI from CKD. Three cut-off points were evaluated: 170 pg/mL, 125 pg/mL, and 132 pg/mL. The 170 pg/mL value showed the highest specificity (88.8%) but the lowest sensitivity (57%), with a positive likelihood ratio (LR+) of 5.1 for identifying CKD. This threshold is particularly useful for confirming CKD, as previously reported [10]. In contrast, the 125 pg/mL and 132 pg/mL cut-offs offered a better balance between sensitivity and specificity-75.3% and 71% sensitivity, and 73.8% and 77.6% specificity, respectively. These thresholds may be more appropriate for excluding CKD in clinical practice. These findings support the diagnostic utility of iPTH in differentiating AKI from CKD using these three cut-off points. A value above 170 pg/mL is best suited for confirming CKD, especially in patients with a high pre-test probability. Values below 125 pg/mL are more useful for ruling out CKD. This highlights iPTH as a potentially valuable diagnostic tool, particularly when clinical history is limited. Distinguishing AKI from CKD is a common challenge in emergency departments, especially in patients without prior medical history. This differentiation is critical, as it influences treatment decisions, prognosis, and the need for interventions such as KRT [5,10,11,15]. Clinicians often rely on clinical and laboratory parameters, including symptom duration, comorbidities, and physical signs such as uremia, anemia, hypocalcemia, hyperphosphatemia, hypertension, or peripheral neuropathy [16-18]. However, specific findings are rare, and additional diagnostic tools are often required. Kidney ultrasound is commonly used to assess kidney size, echogenicity, cortico-medullary differentiation, and the presence of hydronephrosis or cysts [7,19]. Despite its value, ultrasound lacks specificity, and kidney biopsy often remains the only definitive diagnostic option [9]. Because both AKI and CKD can increase iPTH synthesis and secretion, the degree of elevation has been proposed as a distinguishing factor [5,10,11,20]. A systematic review by Roy et al. [5], which included two relevant studies, noted the potential of iPTH in this role. Cavayero et al. described six patients with elevated creatinine and found iPTH to be an accessible and affordable marker for differentiation [11]. Similarly, Ozmen et al. studied 122 patients with creatinine ≥2 mg/dL and reported an AUC of 0.92 for iPTH, with the 170 pg/mL cut-off offering high diagnostic accuracy for CKD [10]. In contrast, the present study found slightly lower performance, likely due to differences in study populations. This study included a broader and less selected population (creatinine ≥1.3 mg/dL) in a university hospital, compared to Ozmen’s specialized setting.

This study has limitations. Its retrospective design introduces a risk of information bias due to missing or inaccurate records. Conducting the study in a high-complexity hospital may limit the generalizability of the findings to other settings. Although inter-rater agreement between the two primary reviewers was almost perfect (κ = 0.81), 9.5% of cases required adjudication by a third reviewer, which may reflect the inherent diagnostic complexity of differentiating AKI from CKD. In addition, the actual prevalence of CKD in our sample (~46.5%) was substantially higher than the 5% anticipated in the sample size calculation, which may have influenced the precision of diagnostic accuracy estimates. Acute biochemical changes during AKI, such as variations in vitamin D, phosphate, and calcium levels, may have influenced iPTH measurements, and we did not perform a dynamic assessment of iPTH at multiple time points for all patients. Finally, while kidney biopsy was used as the reference standard, its invasive nature, high cost, and limited availability reduce its practicality in routine clinical settings.

## Conclusions

iPTH is a promising and well-established biomarker for distinguishing between acute and chronic kidney dysfunction. Its diagnostic utility may vary depending on the clinical context, with different thresholds aiding in the confirmation or exclusion of chronic kidney disease. These findings highlight the potential role of iPTH in emergency and inpatient settings, particularly when medical history is limited or unavailable. Further prospective research is warranted to validate its diagnostic accuracy and define its integration into routine clinical decision-making.
